# The role of sense of coherence in the relationship between posttraumatic stress, depression and anxiety among nurses in South Africa

**DOI:** 10.1177/13591053251408192

**Published:** 2026-01-15

**Authors:** Ashraf Kagee, Bronwyne Coetzee, Phillipa Haine

**Affiliations:** 1 Stellenbosch University, Cape Town, South Africa

**Keywords:** anxiety, depression, nurses, posttraumatic stress, sense of coherence, South Africa

## Abstract

Nurses experience high levels of occupational stress and trauma exposure, placing them at risk for adverse mental-health outcomes. This study examined the relationships between posttraumatic stress symptoms (PTS), Sense of Coherence (SOC), anxiety, and depression among South African nurses. A cross-sectional survey design was employed with 264 nurses from 4 hospitals in the Western Cape province. Participants completed a demographic questionnaire and four self-report measures: the SOC Scale, PTSD Checklist for DSM-5, Beck Anxiety Inventory, and Centre for Epidemiological Studies Depression Scale-Revised. Structural equation modelling assessed whether SOC mediated the effects of PTS on anxiety and depression. Results indicated that SOC partially mediated the relationship between PTS and both anxiety and depression, suggesting that part of the impact of PTS on psychological distress operates through nurses’ SOC levels. These findings highlight the importance of trauma-informed approaches and SOC-enhancing interventions to strengthen nurses’ mental health in high-stress, low-resource, healthcare environments.

## Introduction

Occupational crises in healthcare settings often trigger significant psychological symptoms and behavioural disruptions among affected personnel (Esterwood and Saeed, 2020; Morganstein et al., 2017). Systematic reviews and meta-analyses similarly report disproportionately high levels of insomnia, stress, anxiety, and depression among HCWs compared with the general population (e.g. Blasco-Belled et al., 2022; Ghahramani et al., 2023; Krishnamoorthy et al., 2020; Tong et al., 2022). While these challenges are global, they are particularly pronounced in low- and middle-income countries (LMICs), where healthcare infrastructure and mental-health services are often under-resourced and overstretched (Bong et al., 2020; Deng and Naslund, 2020).

Among HCWs, nurses have borne a disproportionate burden of psychological strain. Often positioned as the first and sometimes sole point of contact, nurses frequently navigate heavy patient loads, limited resources, and repeated exposure to patient suffering and death (Endacott and Blot, 2022; Iddrisu et al., 2023). In South Africa, these challenges are compounded by pre-existing systemic constraints in the public healthcare system, including staff shortages, underfunding, and inconsistent access to psychological support (Harris et al., 2011; Malakoane et al., 2020). Consequently, South African nurses have long demonstrated high rates of emotional exhaustion, compassion fatigue, and symptoms of psychological distress (Payne et al., 2020). Nevertheless, there remains a paucity of research focussing exclusively on South African nurses, as most local studies aggregate nurses with other HCW categories (notable exceptions include Coetzee et al., 2025; Engelbrecht et al., 2021; Haine et al., 2025; Kelly et al., 2022; Onwubu et al., 2023). This gap is concerning given meta-analytic findings that nurses experience significantly higher psychological distress than other HCWs (Al Maqbali et al., 2024).

Among the range of mental-health outcomes observed in nursing populations, posttraumatic stress disorder (PTSD) has emerged as particularly salient (Yuan et al., 2021). Posttraumatic stress disorder (PTSD) is characterised as a psychological response to exposure to actual or threatened death, serious injury, or sexual violence, as defined by both the *Diagnostic and Statistical Manual of Mental Disorders* (fifth ed., text rev.; American Psychiatric Association, 2022) and the *International Classification of Diseases* (11th revision; World Health Organisation, 2019). Within healthcare contexts, secondary or vicarious trauma, resulting from sustained exposure to patients’ suffering and distress, may also fulfil the diagnostic criteria for PTSD (Chen and Chow, 2024; Gabay, 2025). Recent meta-analytic evidence estimates a pooled global prevalence of PTSD at 29% among nurses (*n* = 40 000) across 55 countries (Hernández-Bojorge et al., 2024). Nevertheless, even subclinical manifestations of PTSD have been shown to substantially impair psychological functioning and are associated with increased risk for emotional exhaustion and professional burnout (Bonanno et al., 2011; Galatzer-Levy et al., 2018; Iyadurai et al., 2023).

Beyond PTSD, nurses are also at heightened risk for experiencing symptoms of anxiety and depression, which are frequently comorbid with PTSD and independently associated with burnout, impaired functioning, and workforce attrition (Kisely et al., 2020; Pappa et al., 2020). These mental health indicators warrant focussed attention not only as correlates of PTSD but as critical outcomes in their own right, given their substantial impact on nurses’ wellbeing and healthcare system resilience (Haine et al., 2025). Nevertheless, understanding the mechanisms of trauma responses among nurses and how they relate to psychological outcomes such as depression and anxiety is critical for designing interventions and strengthening workforce sustainability.

One such mechanism is Sense of Coherence (SOC), a central concept in Antonovsky’s (1979, 1987) salutogenic model. This model moves beyond a disease-focussed paradigm and focuses on the processes through which individuals maintain health despite adversity (for a detailed overview of salutogenesis and SOC, see Mittelmark et al., 2017). SOC is defined as a global orientation reflecting an individual’s capacity to perceive life as comprehensible, manageable, and meaningful (Antonovsky, 1987). SOC influences how individuals interpret adversity, mobilise coping resources, and sustain psychological wellbeing (Masanotti et al., 2020). SOC uniquely combines relevant aspects of behavioural, cognitive and motivational resistance (Mittelmark et al., 2017) and thus offers an advantage over other factors that may protect an individual from the negative effects of adversity (e.g. self-efficacy and hardiness). Importantly, within clinical contexts, the SOC concept further comprises meaning-making, which is one of the core components in established PTSD treatments (Schnyder et al., 2015).

A meta-analysis of 45 studies (*n* = 10,883) revealed a significant negative correlation between SOC and PTSD symptom severity, in which higher SOC levels are associated with lower PTSD symptom severity (Schäfer et al., 2019). However, since all but one of the included studies were cross-sectional, this evidence reflects a correlational snapshot rather than clear temporal or causal direction. Schäfer et al. (2019) thus caution that severe trauma may reduce SOC, or conversely, that lower SOC may increase vulnerability to PTSD symptoms. The authors also note that co-occurring conditions, such as depression or general distress, may further explain this relationship. Similarly, Veronese and Pepe (2014) examined SOC as a determinant or component of psychological distress among Palestinian health providers (*n* = 218). SOC was found to be a determinant of the relationship between trauma and anxiety, social dysfunction, and loss of confidence. Mediation analysis confirmed that SOC partially mediated the impact of trauma on both anxiety and social dysfunction whereas it fully mediated the relationship between trauma and loss of confidence (Veronese and Pepe, 2014). Accordingly, examining whether SOC helps account for the relationship between posttraumatic stress (PTS) symptoms and psychological distress (e.g. anxiety and depression) among HCWs may refine theoretical understanding and inform intervention design in high-stress healthcare environments.

Research on SOC among nurses in LMICs, and in African contexts specifically, remains limited (Masanotti et al., 2020). Although the small body of South African literature indicates that SOC is positively associated with adaptive coping and psychological resilience in nursing populations (Cilliers, 2003; Coetzee et al., 2025; Levert et al., 2000; Van der Colff and Rothmann, 2009), few studies have investigated its potential mediating role in the context of HCWs and trauma exposure. Specifically, to the best of our knowledge, no research to date has examined whether SOC mediates the relationship between PTS symptoms and anxiety and depression, among South African nurses. This study therefore addresses this gap by examining whether SOC mediates the relationship between PTS symptoms and symptoms of anxiety and depression among South African nurses working within high-stress healthcare environments.

## Methods

### Participants and procedure

This cross-sectional study employed a convenience sample of 264 registered nurses from 4 hospitals in the Western Cape province of South Africa. Data were collected from April 2022 to May 2023. Recruitment notices were displayed in hospitals, inviting interested nurses to contact the research team via WhatsApp. Participants then received a link to an online survey, which included the informed consent form and questionnaire battery. Alternatively, nurses were given the option to complete a paper-based version, returned anonymously via sealed drop-boxes in staff break rooms. Participation was voluntary and anonymous. The survey took approximately 20 minutes to complete.

### Instruments

Participants completed a short demographic questionnaire and a battery of four self-report instruments as follows:

*Demographic data* were collected on age, gender, working sector, education level attained, employment type and marital status.
*Sense of Coherence* (SOC) was assessed with the 13-item SOC scale (Antonovsky, 1993), which includes 3 subscales: Meaningfulness (4 items), Comprehensibility (5 items), and Manageability (4 items). Items are rated on a seven-point Likert scale, yielding a total score from 13 (low SOC) to 91 (high SOC). Prior studies report strong reliability for the 13-item version (α = 0.74–0.91; Söderhamn and Holmgren, 2004) and recent validation among South African samples (Kagee et al., 2024; Padmanabhanunni and Pretorius, 2025).
*Posttraumatic stress* (PTS) was assessed with the Posttraumatic Stress Disorder-Checklist for DSM-5 (PCL-5: Blevins et al., 2015). The PCL-5, a self-report measure comprising 20 items, assesses the symptoms of PTSD as delineated in the DSM-5 (Blevins et al., 2015). The PCL-5 serves as a screening tool across various settings to identify potential PTSD cases (e.g. Blevins et al., 2015; Hall et al., 2019). The PCL-5 demonstrates robust internal consistency (α = 0.95; Hall et al., 2019) and utility in South African samples (Kagee et al., 2022). Although the PCL-5 does not assess trauma tied to a specific event, it serves as a validated screening tool for posttraumatic stress symptoms across diverse occupational and healthcare contexts (see Hernández-Bojorge et al., 2024), making it appropriate for this study.
*Anxiety* was measured with the 21-item Beck Anxiety Inventory (BAI; Beck et al., 1988), which assesses somatic and cognitive aspects of anxiety. Items are rated on a four-point scale (1 = Not at all to 4 = Severely), yielding total scores from 0 to 63. Strong internal consistency has been consistently demonstrated by the BAI, with alpha coefficients exceeding 0.90 reported globally, including Korea (Lee et al., 2018) and the United States (Chapa, 2022). Similarly, when applied in the South African setting, the BAI has shown satisfactory internal consistency reliability (Kagee et al., 2015).
*Depression* was assessed with the Centre for Epidemiological Studies Depression Scale Revised (CESD-R: Eaton et al., 2004). The CESD-R is a self-report measure consisting of 20 items designed to assess 9 categories of depression symptoms, as outlined in the DSM-5. Participants rate each item on a four-point Likert scale from 0 to 4. The CESD-R demonstrates favourable psychometric attributes, showing robust internal consistency along with efficacious convergent and divergent validity (Van Dam and Earleywine, 2011). In South Africa, the CESD-R has been effectively employed, yielding outcomes with good effect (e.g. Hassem, 2022; Kagee et al., 2020).

### Data analysis

All data analyses were conducted using IBM SPSS Statistics for Windows, Version 28 (IBM Corp, 2021) and R (R Core Team, 2025) with the *seminr* package (Version 2.3.4; Ray et al., 2021). Descriptive statistics were computed for all variables, and associations between demographic variables and outcomes were examined using Pearson correlations and Mann-Whitney U tests, as appropriate. To address the study aims, mediation analyses were performed using partial least squares structural equation modelling (PLS-SEM), which was deemed suitable given the inclusion of multiple latent constructs and the need to examine both direct and indirect effects within a single model. Additionally, we conducted hierarchical multiple regression analyses to determine the unique contribution of PTS and SOC to variance in anxiety and depression scores, above and beyond demographic covariates. Relevant demographic variables (age, gender, working sector, education level, employment type, and marital status) were entered in Block one, PTS symptoms in Block two, and SOC in Block three, to examine their unique contributions. Variance Inflation Factor (VIF) values were all within acceptable limits (mean = 1.14, all <5.0), indicating no significant multicollinearity. Clinically significant symptom levels were classified using the following cutoffs: PCL-5 ⩾ 31 for severe PTS (Kagee et al., 2022); CES-D ⩾ 16 for clinically significant depression and ⩾27 for elevated severity (Van Dam and Earleywine, 2011); and BAI ⩾ 26 for severe anxiety (Beck et al., 1988).

### Ethical considerations

This research received ethical clearance from the Health Research Ethics Committee at Stellenbosch University, with reference number N21/05/012-COVID-19. Participation in the study was voluntary and anonymous. As a gesture of appreciation, participants received a R100 grocery voucher redeemable at Shoprite. Before commencing the online survey, participants were required to provide informed consent through the survey’s landing page, or through signed written consent forms for those who opted to complete the paper-based version. Contact details for free counselling services were provided to all participants in case of distress related to survey participation.

## Results

### Description of the sample

The final sample comprised 264 nurses, primarily women (82%), with a mean age of 34.40 years (SD = 7.90). Most participants worked in the public sector (76%), held full-time employed positions (90%), and had completed post-school education (88%). Just over half were married or partnered (54%).

### Clinical scores

Clinically significant depressive symptoms (CES-D ⩾ 16) were reported by 56% of participants, with 32% showing elevated depressive symptoms (CES-D ⩾ 27). Severe anxiety symptoms (BAI ⩾ 26) were present in over 32%, and 39% reported clinically significant posttraumatic stress (PTS) symptoms (PCL-5 ⩾ 31). The mean SOC score was 54.9 (SD = 11.1), with scores ranging from 16 to 85, indicating moderate levels of sense of coherence among participants.

### Bivariate correlations

Descriptive statistics, internal consistencies, and correlations are presented in Table 1. Internal consistency reliabilities were acceptable, with Cronbach’s alpha values ranging from 0.80 to 0.96. PTS symptoms showed significant positive correlations with anxiety (*r* = 0.57, *p* < 0.001) and depression (*r* = 0.55, *p* < 0.001), and a significant negative correlation with SOC (*r* = −0.26, *p* < 0.001). Age was negatively correlated with anxiety (*r* = −0.23, *p* < 0.001), depression (*r* = −0.19, *p* < 0.001), and PTS (*r* = −0.22, *p* < 0.001), and was weakly positively correlated with SOC (*r* = 0.15, *p* < 0.05).

**Table 1.** Descriptive statistics, reliabilities and intercorrelations.

Variables	1	2	3	4	5	Mean	SD	Alpha
1. PTS	—					27.17	18.61	0.96
2. SOC	−0.26**	—				54.92	11.14	0.80
3. Anxiety	0.57**	−0.28**	—			18.04	14.81	0.96
4. Depression	0.55**	−0.27**	0.80**	—		20.50	15.68	0.95
5. Age	−0.22**	0.15*	−0.23**	−0.19**	—	34.4	7.99	

PTS: posttraumatic stress; SOC: sense of coherence.

* *p* < 0. 05. **p < 0.01.

### Demographic factors

A Mann-Whitney *U* test showed no significant gender differences in psychological outcomes (see Supplemental Table S1). However, PTS symptoms differed by working sector, with private-sector nurses reporting higher levels than public-sector nurses, *U* = 6183.50, *p* = 0.008, *r* = 0.17 (see Supplemental Table S2).

### Mediation model

**Figure 1.**
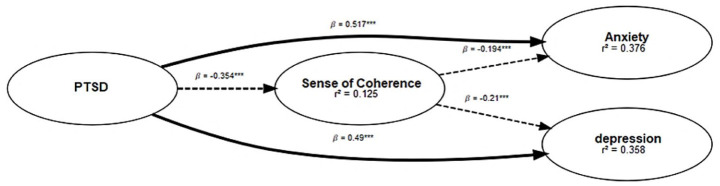
Partial least squares structural equation model (PLS-SEM) illustrating the interrelationships among posttraumatic stress (PTS), sense of coherence (SOC), anxiety, and depression. This figure represents the inner (structural) model of the SEM, depicting the directional relationships among latent constructs. Solid arrows represent direct effects, while dotted arrows indicate indirect (mediated) paths via SOC. Regression weights are standardised (***p < 0.001, **p < 0.01, *p < 0.05). The outer loadings for the measurement model are provided in the Supplemental Material (Supplemental Figure S1).

Figure 1 shows the inner (structural) model of the PLS-SEM tested. The outer loadings for the measurement model are provided in the Supplemental Material (see Supplemental Figure S1). For assessing internal consistency, composite reliability values greater than 0.70 were deemed indicative of good reliability (Hair et al., 2019). In this study, composite reliability values ranged between 0.85 and 0.96. Convergent validity was evaluated through average variance extracted (AVE) values. All of the AVE values were above the guideline level of 0.5 (Mantsos et al., 2024). Discriminant validity was checked using heterotrait/monotrait ratios and were all found to be acceptable (Kline, 2023). All outer loadings of the manifest variables were statistically significant (*p* < 0.001) and ranged between 0.57 and 0.85. Age and working sector were included as covariates to control for potential confounds. The results of the mediation analysis are reported in Table 2.

**Table 2.** The mediating role of sense of coherence (SOC).

Effect	Standardised path coefficient (β)	95% CI	*t* statistic	*p*
Direct effects				
PTS → SOC	−0.35	[−0.46, −0.27]	−7.13	<0.001
PTS → anxiety	0.52	[0.4, 0.62]	9.01	<0.001
PTS → depression	0.49	[0.38, 0.59]	9.19	<0.001
SOC → anxiety	−0.19	[−0.29, −0.11]	−4.14	<0.001
SOC → depression	−0.21	[−0.31, −0.12]	−4.57	<0.001
Indirect effects				
PTS → SOC → anxiety	0.07	[0.04, 0.11]	3.51	<0.001
PTS → SOC → depression	0.07	[0.04, 0.13]	3.53	<0.001

PTS: posttraumatic stress; SOC: sense of coherence.

PTS symptoms were significantly negatively associated with SOC (β = −0.35, *p* < 0.001), and significantly positively associated with anxiety (β = 0.52, *p* < 0.001) and depression (β = 0.49, *p* < 0.001). Similarly, SOC was significantly negatively associated with anxiety (β = −0.19, *p* < 0.001) and depression (β = −0.21, *p* < 0.001). Significant relationships were found for the mediating relationship of SOC between PTS and anxiety (β = 0.07; *p* < 0.001) and between PTS and depression (β = 0.07; *p* < 0.001).

### Regression diagnostics

Regression diagnostics indicated no violations of assumptions (e.g. acceptable Cook’s distances, Mahalanobis distances, and variance inflation factors).

### Predictors of anxiety symptoms

Table 3 summarises the regression models. For anxiety, the final model explained 35.9% of the variance (*R*^2^ = 0.359), indicating a large effect size (Cohen, 1988). Adding PTS symptoms significantly increased explained variance (*ΔR*^2^ = 0.274, *p* < 0.001). SOC added a small, marginally non-significant contribution (*ΔR*^2^ = 0.010, *p* = 0.059). As illustrated in the Supplemental Table S3, PTS symptoms (β = 0.53, *p* < 0.001) and marital status (β = 0.15, *p* = 0.011) were significant predictors.

**Table 3.** Model summary with anxiety and depression scores as criterion variables.

Outcome	Model	Predictors	*R*	*R* square	Adj *R* square	*R* square change	*F* ratio	*F* change
Anxiety symptoms	1	Demographic	0.273	0.074	0.054	0.074	3.638	0.003**
	2	Demographic, PTS	0.590	0.348	0.331	0.274	94.576	<0.001**
	3	Demographic, PTS and SOC	0.599	0.359	0.339	0.010	3.614	0.059
Depressive symptoms	1	Demographic	0.249	0.062	0.041	0.062	2.986	0.012*
	2	Demographic, PTS	0.570	0.325	0.307	0.263	87.723	<0.001**
	3	Demographic, PTS and SOC	0.575	0.330	0.309	0.005	1.745	0.188

*Note*. Demographic variables: age, gender, working sector, education, employment, marital status.

PTS: posttraumatic stress; SOC: sense of coherence.

** *p* < 0.01. *p < 0.05.

### Predictors of depressive symptoms

For depression, the final model explained 33.0% of the variance (*R*^2^ = 0.330), indicating a large effect size (Cohen, 1988). Adding PTS symptoms again accounted for the largest increase (Δ*R*^2^ = 0.263, *p* < 0.001), while SOC did not add significant unique variance (Δ*R*^2^ = 0.005, *p* = 0.188). As illustrated in the Supplemental Table S4, PTS symptoms (β = 0.52, *p* < 0.001) and education (β = −0.16, *p* = 0.016) were significant predictors.

## Discussion

Promoting the mental health of nurses working under sustained occupational stress is essential to safeguard wellbeing and ensure the continued delivery of high-quality patient care. Consistent with global evidence (Al Maqbali et al., 2021, 2024), our results revealed high levels of psychological distress among South African nurses. Over half the sample reported clinically significant depressive symptoms, while nearly one-third indicated severe anxiety and PTS symptoms. These figures demonstrate the toll of persistent occupational stressors and exposure to trauma within an already strained healthcare system (Harris et al., 2011; Malakoane et al., 2020).

Using PLS-SEM, we modelled PTS, SOC, anxiety, and depression as latent constructs derived from multiple indicators, allowing robust estimation of both measurement reliability and structural relationships. Consistent with prior research, PTS symptoms emerged as a strong predictor of both anxiety and depression (Andhavarapu et al., 2022; Mosheva et al., 2021; Seto et al., 2020); wherein cumulative exposure to patient death and suffering may be considered significant traumatic stressors within this population. This pattern aligns with network theory (Borsboom, 2017; Fried et al., 2017), which suggest that overlapping symptoms such as hyperarousal and intrusive thoughts may reinforce one another, creating feedback loops of distress. Neurobiological explanations further point to dysregulated hypothalamic-pituitary-adrenal (HPA) axis functioning and stress hormone imbalances (Brady et al., 2000; Kamin and Kertes, 2017) as pathways through which traumatic stress can simultaneously fuel anxiety and depressive symptoms. Psychologically, trauma-induced hypervigilance, avoidance, and loss of meaning may undermine social support and coping self-efficacy (Campbell and Renshaw, 2018; Gariépy et al., 2016), intensifying vulnerability to comorbid psychopathology.

Crucially, our PLS-SEM demonstrated that SOC partially mediates the relationship between PTS symptoms and both anxiety and depression. This result suggests that a significant portion of the impact of PTS on nurses’ mental health is explained through their sense of comprehensibility, manageability, and meaningfulness (i.e. SOC). This finding aligns with Antonovsky’s (1987) salutogenic model. Our findings further mirror those of Veronese and Pepe (2014), who found that SOC partially mediates the relationship between traumatic stress and anxiety and social difficulties among Palestinian health professionals working in a chronic conflict zone.

The hierarchical regression analysis supports these findings by showing that PTS symptoms explained the largest proportion of variance in both anxiety and depression, even after demographic covariates were controlled for. Although SOC was significantly associated with lower anxiety and depression in bivariate models, its additional predictive contribution became small and non-significant when entered alongside PTS in the regression blocks. Our findings suggest that while SOC functions as an important pathway through which PTS shapes psychological distress, the strong direct effects of PTS appear to overshadow SOC’s unique predictive influence when both variables are considered simultaneously. This pattern aligns with salutogenic theory, which recognises that although SOC supports adaptive coping, chronic or high-intensity stressors can temporarily overshadow its protective influence when demands exceed available resistance resources (Antonovsky, 1987; Mittelmark et al., 2017). Contemporary salutogenic perspectives further emphasise that SOC promotes well-being through both direct and indirect pathways by enhancing meaningfulness, manageability, and comprehensibility. Accordingly, rather than implying that SOC’s primary role lies solely in mediating trauma effects, our findings suggest that SOC may exert both direct and indirect influences on nurses’ mental health outcomes.

Notably, SOC was positively associated with age, suggesting that older nurses may build stronger coping capacities through accumulated experience (Nilsson et al., 2010; Wiesmann and Hannich, 2019). Conversely, younger nurses, who are often less experienced, may be less equipped to make sense of traumatic stressors or believe in their capacity to manage them, leaving them more vulnerable to developing comorbid mental health conditions. This result points to the importance of targeting SOC-building initiatives towards early-career nurses. As such, our findings suggest that younger nurses should be considered a particularly vulnerable subgroup and prioritised for targeted trauma-informed support and SOC-building interventions. Unexpectedly, nurses working in the private sector reported significantly higher PTS symptom scores than those in the public sector. This finding contrasts with common assumptions that public sector nurses, who face chronic underfunding, staff shortages, and overcrowding (Harris et al., 2011; Malakoane et al., 2020), would exhibit greater distress. One explanation may be that private-sector nurses face distinct stressors, such as increased performance pressure, heightened patient expectations, or ethical tensions linked to resource allocation, which may amplify moral injury and emotional labour (Williamson et al., 2020). Alternatively, habituation to adversity and crisis may partly blunt psychological reactivity among public sector nurses who routinely work under strained conditions. This finding highlights the importance of sector-specific mental health strategies that account for the complex, context-dependent drivers of distress rather than focussing solely on resource comparisons.

Finally, the regression analyses indicated that certain demographic variables, specifically marital status and education level, were modest but significant predictors of psychological distress. Although these variables were not the central focus of the study, the findings lend support to prior research by Haine et al. (2025), which suggests that psychosocial factors such as age, marital status, and education level may influence nurses’ vulnerability to poor mental health outcomes in the South African context. These results emphasise the importance of considering individual-level characteristics when designing mental health interventions.

### Clinical implications

This study offers several practical implications. First, the high rates of PTS, anxiety, and depression emphasise the critical need for routine trauma-informed screening, early intervention, and referral pathways within healthcare settings. Given the limited mental health infrastructure in South Africa (Sorsdahl et al., 2023), scalable solutions such as mobile or e-mental health tools could help reach more nurses in need (Drissi et al., 2021; Fiol-DeRoque et al., 2021). Second, our finding that SOC partially explains how trauma affects distress suggests that interventions should aim to strengthen comprehensibility, manageability, and meaning-making among nurses. Practical avenues include peer-support groups, supervisor-led check-ins, and reflective debriefing sessions to help nurses make sense of stressors, build coping skills, and reconnect with meaningful aspects of their work. Such approaches may be particularly impactful for younger nurses, who showed lower SOC levels. Finally, the unexpected finding of higher PTS symptoms among private sector nurses and the influence of individual demographic factors highlight the need for sector-specific, tailored mental health strategies that address the unique workplace demands and personal circumstances influencing nurses’ wellbeing. As with all studies, there are limitations that warrant attention. First, the current study’s cross-sectional design precludes causal conclusions; temporal precedence cannot be established, and the directionality of relationships between PTS, SOC, depression and anxiety could be bidirectional. Second, self-report measures may be subject to social desirability bias. Third, voluntary participation may have introduced self-selection bias. Finally, the sample was limited to 4 hospitals in a country with over 600 healthcare facilities, which limits generalisability. Future longitudinal and mixed-methods studies are needed to clarify causal pathways and inform tailored interventions.

## Conclusion

This study adds to growing evidence on the mental health burden borne by nurses in resource-constrained healthcare systems and highlights the central role of trauma in shaping emotional wellbeing. Crucially, it identifies SOC as a mechanism through which posttraumatic stress influences broader mental health outcomes, offering a potential target for preventative and therapeutic interventions. Strengthening psychological resources such as SOC and mitigating trauma-related distress, particularly among younger and private-sector nurses, may be key to promoting resilience, reducing distress, and safeguarding the integrity of healthcare delivery.

## Supplemental Material

sj-docx-1-hpq-10.1177_13591053251408192 – Supplemental material for The role of sense of coherence in the relationship between posttraumatic stress, depression and anxiety among nurses in South Africa Supplemental material, sj-docx-1-hpq-10.1177_13591053251408192 for The role of sense of coherence in the relationship between posttraumatic stress, depression and anxiety among nurses in South Africa by Ashraf Kagee, Bronwyne Coetzee and Phillipa Haine in Journal of Health Psychology
