# Supplementary material for: The role of sense of coherence in the relationship between posttraumatic stress, depression and anxiety among nurses in South Africa
Source: J Health Psychol. 2026 Jan 15;31(9):3898–911. doi: 10.1177/13591053251408192 (PMC13365323; doi:10.1177/13591053251408192)
Supplement: sj-docx-1-hpq-10.1177_13591053251408192 – Supplemental material for The role of sense of coherence in the relationship between posttraumatic stress, depression and anxiety among nurses in South Africa [file sj-docx-1-hpq-10.1177_13591053251408192.docx]

**Supplementary Material**

**Table S1**

Mann-Whitney U test results for gender

| Variable | Gender | *n* | Sum of Ranks | *U* | Sig. (adjusted for ties) |
| --- | --- | --- | --- | --- | --- |
| Anxiety | Women | 216 | 28123.50 | 4687.50 | .41 |
|  | Men | 47 | 6592.50 |  |  |
| PTS | Women | 216 | 28182.00 | 4746.00 | .49 |
|  | Men | 47 | 6534.00 |  |  |
| SOC | Women | 216 | 131.91 | 5055.500 | .97 |
|  | Men | 47 | 132.44 |  |  |
| Depression | Women | 216 | 28194.00 | 4758.00 | .50 |
|  | Men | 47 | 6522.00 |  |  |

*Note*: PTS = Posttraumatic Stress; SOC = Sense of Coherence. ^**^*p* < .01, ^*^*p* <. 05

**Table S2**

Mann-Whitney U test results for working sector

| Variable | Education | *n* | Sum of Ranks | *U* | Sig. (adjusted for ties) |
| --- | --- | --- | --- | --- | --- |
| Anxiety | Government | 176 | 112.12 | 5875.000 | .05 |
|  | Private | 57 | 132.07 |  |  |
| PTS | Government | 176 | 110.37 | 6183.500 | .008** |
|  | Private | 57 | 137.48 |  |  |
| SOC | Government | 176 | 118.04 | 4833.000 | .68 |
|  | Private | 57 | 113.79 |  |  |
| Depression | Government | 176 | 114.70 | 5421.100 | .36 |
|  | Private | 57 | 124.11 |  |  |

*Note*: PTS = Posttraumatic Stress; SOC = Sense of Coherence. ***p* < .01, ^*^*p* <. 05

**Table S3**

Predictors of anxiety symptoms (Beck Anxiety Inventory) (*n* = 264)

|  |  | Unstandardised Coefficients | | Standardised Coefficients | |  |
| --- | --- | --- | --- | --- | --- | --- |
| Model | | *B* | Standard Error | Beta | *t* | Sig. |
|  | Constant | 38.628 | 15.323 |  | 2.521 | .012 |
|  | Age | -0.032 | 0.110 | -0.017 | -0.295 | .768 |
|  | Working Sector | 0.421 | 1.884 | 0.012 | 0.233 | .823 |
|  | Education | -4.162 | 2.273 | -0.114 | -1.831 | .068 |
|  | Employment | -1.155 | 1.806 | -0.040 | -0.640 | .523 |
|  | Marital Status | 1.546 | 0.600 | 0.147 | 2.577 | .011* |
|  | PTS symptoms | 0.412 | 0.044 | 0.530 | 9.365 | <.001** |
|  | SOC | -0.145 | 0.076 | -0.106 | -1.901 | .059 |

*Note*: PTS = Posttraumatic Stress; SOC = Sense of Coherence. ***p* < .01, **p*< .05

**Table S4**

Predictors of depressive symptoms (Center for Epidemiological Studies-Depression Scale, CESD) (*n* = 264)

|  | | Unstandardised Coefficients | | Standardised Coefficients | |  |
| --- | --- | --- | --- | --- | --- | --- |
| Model | | *B* | Standard Error | Beta | *t* | Sig. |
|  | Constant | 47.635 | 16.473 |  | 2.892 | 0.004 |
|  | Age | 0.051 | 0.118 | 0.025 | 0.430 | 0.668 |
|  | Working Sector | -1.553 | 2.025 | -0.43 | -0.767 | 0.444 |
|  | Education | -5.928 | 2.444 | -0.155 | -2.426 | 0.016* |
|  | Employment | 0.351 | 1.942 | 0.012 | 0.181 | 0.857 |
|  | Marital Status | 1.235 | 0.645 | 0.112 | 1.915 | 0.057 |
|  | PTS symptoms | 0.428 | 0.047 | 0.524 | 9.061 | < .001** |
|  | SOC | -0.108 | 0.082 | -0.075 | -1.321 | 0.188 |

*Note*: PTS = Posttraumatic Stress; SOC = Sense of Coherence. ***p* < .01, * *p*< .05


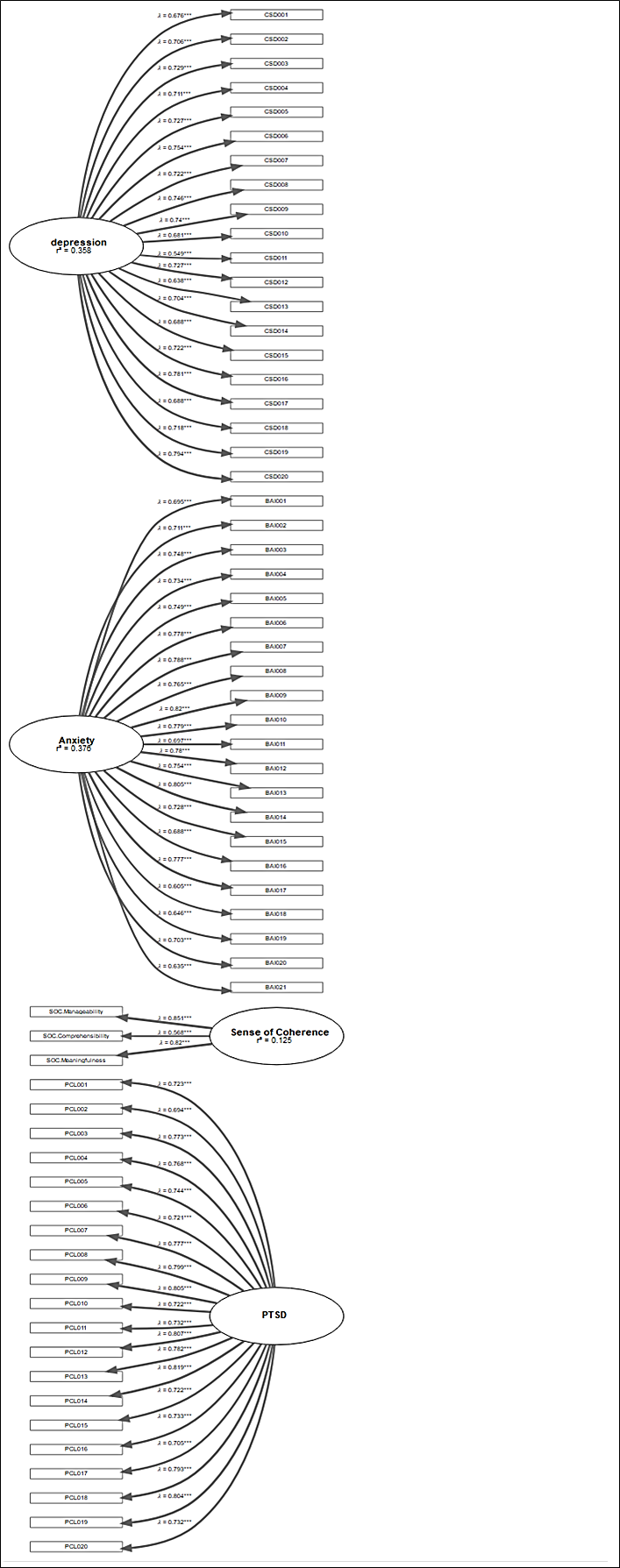


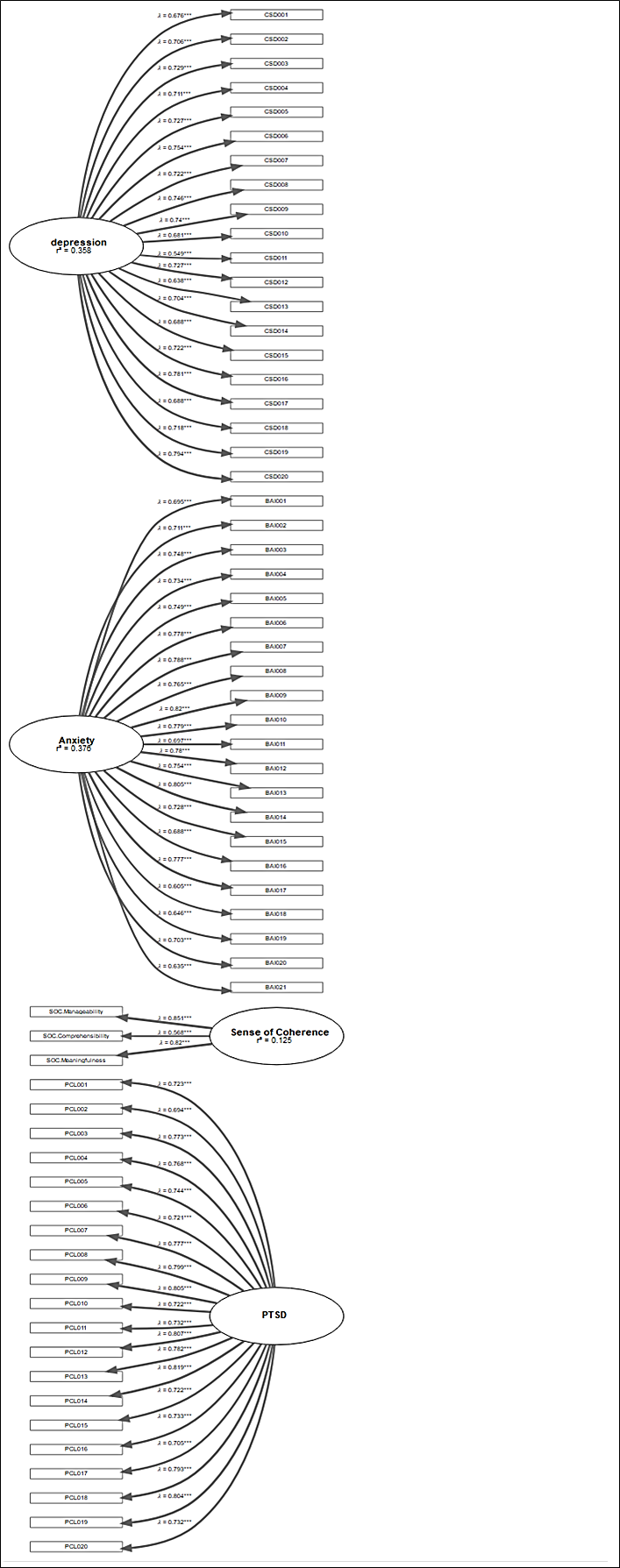


**Figure S1.** Outer loadings of the measurement model for posttraumatic stress (PTS), sense of coherence (SOC), anxiety, and depression. All loadings are significant (***p* < .001) and meet reliability thresholds (> 0.50).
